# Gamified Formative Assessment in a First-Year Medical Morphology Course: Qualitative Study

**DOI:** 10.2196/90651

**Published:** 2026-10-22

**Authors:** Álvaro Herrera Alcaíno, Sofía Paz Araya Figueroa, Valentina Francisca Riquelme Vilches, Eduardo Vargas Zurita, Renata Valentina Cozzi Ahumada, Camila Riquelme Bahamondes, Carlos Alberto Rosas Chuñil

**Affiliations:** 1 Faculty of Medicine, San Sebastián University, Lota 2465, Providencia, Santiago, Metropolitana, 7500000, Chile, 56 222606000; 2 Faculty of Medicine, San Sebastián University, Valdivia, Los Ríos, Chile; 3 Faculty of Medicine, San Sebastián University, Concepción, Biobío, Chile

**Keywords:** medical education, formative assessment, gamification, Kahoot!, qualitative research, Technological Pedagogical Content Knowledge, TPACK, morphology, anatomy

## Abstract

**Background:**

Formative assessment plays a central role in medical education by offering low-risk practice and timely feedback, particularly in visually and practically demanding courses, such as morphology. Gamified digital tools can foster active learning; however, their educational value relies on pedagogical integration rather than technology alone. The Technological Pedagogical Content Knowledge (TPACK) model provides a framework for understanding this relationship.

**Objective:**

This study explored students’ and a teacher’s perceptions of Kahoot! as a gamified formative assessment tool in a first-year medical morphology course, identifying facilitators, tensions, and perceived educational value through the TPACK framework.

**Methods:**

This qualitative, exploratory study adopted an interpretive phenomenological orientation. It was conducted within the framework of the RAÍCES 2 (Red de ayudantes de investigación comprometidos con la educación en salud; Network of Student Researchers Committed to Health Professions Education) educational research project at Universidad San Sebastián (Valdivia, Chile). Kahoot! was implemented at the end of class sessions to simulate the visual and time demands of authentic practical examinations. Data collection included 2 online focus groups with first-year medical students (n=14) and 1 semistructured interview with the course teacher (n=1). Transcripts were analyzed using deductive coding informed by the TPACK framework and the project’s analytical axes, combined with reflexive thematic analysis following Braun and Clarke. ATLAS.ti was used to support the analysis.

**Results:**

Six interrelated themes emerged: (1) techno-disciplinary alignment, reflecting the value of authentic, high-resolution anatomical images and time constraints mirroring practical examinations; (2) Kahoot! as a formative closure, serving as an exit check that guided self-assessment and the identification of learning gaps; (3) from score to learning, highlighting the importance of immediate postquestion instructor feedback explaining both correct and incorrect answers; (4) motivation and socioemotional climate, whereby gamification enhanced engagement but triggered evaluative anxiety and leaderboard frustration, which were buffered by participant anonymity; (5) implementation conditions, mediated by connectivity, device availability, and time allocation; and (6) projection and improvement, highlighting the need for institutional question repositories and systematic curriculum integration.

**Conclusions:**

Kahoot! effectively functions as a formative assessment strategy when intentionally aligned with disciplinary and practical evaluative demands. Its educational value is not inherent to the software but is determined by explicit pedagogical mediation and careful attention to students’ socioemotional dynamics.

## Introduction

### Formative Assessment and Gamification in Medical Education

In medical education, formative assessment plays a key role by allowing students to practice in academically low-risk environments and to receive timely feedback. These opportunities are particularly relevant for preparing students for high-stakes assessments, such as anatomical structure recognition exercises, which are common in courses with a strong practical and visual component.

In this context, the use of digital strategies for formative purposes has steadily increased in medical education. A recent review published in JMIR Medical Education highlights the expansion of digital teaching [1], noting that gamification tools, such as web-based quizzes, can help synthesize essential content [2] and support engaging synchronous learning activities for students [3]. Available evidence suggests that, when appropriately implemented, these tools are associated with higher levels of participation and satisfaction, thereby supporting learning processes.

### Anatomical Training Demands and the Technological Pedagogical Content Knowledge Framework

In September 2025, an educational activity was implemented at Universidad San Sebastián (Valdivia, Chile) for first-year medical students enrolled in the morphology I course. The intervention consisted of an end-of-class formative assessment activity using the gamification tool Kahoot! It was intentionally designed to simulate the specific conditions, time constraints, and pressure encountered during authentic practical assessments in the course, such as gymkhanas (station-based examinations focused on timed anatomical structure identification).

Although Kahoot! is widely used for reviewing cross-cutting content [2], its effectiveness largely depends on context and pedagogical design. Previous studies have described this platform as a motivating tool in health professions education [3,4]; however, its impact does not reside solely in the technology itself, but rather in how it is pedagogically integrated. From the theoretical perspective of Technological Pedagogical Content Knowledge (TPACK), the effective use of educational technologies requires a balanced articulation among technology, pedagogy, and disciplinary content [5,6].

Despite its widespread use, evidence about how this type of tool is experienced in highly visual disciplines, such as morphology, remains limited. In particular, there is limited understanding of students’ and teachers’ perceptions regarding its contribution to learning, as well as the facilitators and barriers to its implementation. This study explored students’ and a teacher’s experiences with Kahoot! as a formative assessment tool in morphology I. Using qualitative methods and the TPACK framework, we identified implementation barriers, facilitators, and perceived learning outcomes.

In addition, this study extends beyond describing participants’ perceptions by identifying pedagogical conditions and transferable design principles for gamified formative assessment in visually intensive medical courses. Interpreting these findings through the TPACK framework provides guidance for designing technology-enhanced formative assessment activities that align disciplinary content, pedagogy, and digital tools.

The aim of this study was to explore students’ and the teacher’s perceptions regarding the use of Kahoot! as a gamified formative assessment tool in a first-year medical morphology course. Specifically, this study sought to identify the facilitators, tensions, and perceived educational value of this tool when interpreted through the TPACK framework. Ultimately, we intend to provide transferable pedagogical conditions and design principles to guide educators in integrating technology-enhanced assessments within visually intensive medical disciplines.

## Methods

### Study Design and Context

This study followed a qualitative, exploratory design informed by an interpretive phenomenological orientation, which guided the study’s focus on understanding participants’ lived experiences and the meanings they attributed to the use of Kahoot! within the morphology course. Rather than adopting a specific phenomenological method, this orientation served as an epistemological framework to support an interpretive analysis of participants’ accounts. The study was conducted within the framework of the RAÍCES 2 (Red de ayudantes de investigación comprometidos con la educación en salud; Network of Student Researchers Committed to Health Professions Education) project (2025) and reports findings derived from an educational intervention implemented in the morphology I course in the Medicine program at Universidad San Sebastián, Valdivia campus, Chile. Data collection took place in September 2025.

### Participants and Recruitment

Participants were first-year medical students enrolled in the morphology I course, which had a total enrollment of 76 students during the intervention period. A voluntary sampling strategy was used. Inclusion criteria were as follows: (1) enrollment in the Medicine program at Universidad San Sebastián, Valdivia campus; (2) registration in the morphology I course in September 2025; and (3) participation in the Kahoot!-based educational activity. No exclusion criteria were applied.

After the intervention, students who met the inclusion criteria were invited in person during class sessions to participate in the study. In addition, the invitation was disseminated through social media, along with a link to a participation form. In total, 14 students agreed to participate, representing approximately 11% of the eligible student cohort. As participation was voluntary, this proportion reflects a self-selected sample of students willing to share their experiences.

Given the qualitative and exploratory nature of the study, the sample was considered adequate for the study’s aims, providing sufficient depth and diversity of experiences to support an interpretive understanding of participants’ perspectives. Consistent with qualitative research, emphasis was placed on the richness and relevance of the data rather than on statistical representativeness.

Prior to data collection, participants were invited to attend an in-person meeting at Universidad San Sebastián, Valdivia campus, during which the objectives of the study were explained and written informed consent was obtained. The volunteers were then assigned to 2 focus groups, each composed of 7 students. In addition, an individual semistructured interview was conducted with the course teacher, who was responsible for implementing the intervention, to complement the students’ perspectives.

### Data Collection

Data collection was conducted remotely in September 2025 through synchronous sessions held via Microsoft Teams. Two focus groups with students and 1 individual interview with the course teacher were held.

All sessions were semistructured and guided by interview protocols developed in advance by the research team. Separate guides were designed for students and for the teacher in order to capture their distinct perspectives.

The student focus group guide explored topics such as perceptions of Kahoot! as a formative assessment tool, its influence on learning and motivation, facilitators and barriers to its use, and its alignment with the visual and evaluative demands of the course.

The teacher interview guide focused on pedagogical intentions, implementation decisions, perceived educational value, challenges encountered, and reflections on the integration of technology, pedagogy, and content. The development of both guides was informed by the TPACK framework, ensuring alignment with the study’s theoretical perspective. The full interview and focus group guides are provided in Multimedia Appendix 1.

Each focus group was moderated by a member of the research team. The first focus group was facilitated by EVZ, the second by VFRV, and the teacher interview was conducted by SPAF. None of the moderators had a direct teaching relationship with the participants in order to minimize potential biases related to academic hierarchies. The sessions lasted approximately 60 minutes. No field notes were taken. With participants’ informed consent, all sessions were audio recorded for subsequent analysis. The recordings were transcribed verbatim; only the transcription of the first focus group was supported by AI-assisted tools. Subsequently, all transcripts were manually reviewed and corrected by members of the research team to ensure accuracy and fidelity to the original recordings.

### Data Analysis

The analysis was conducted within an interpretive qualitative framework consistent with the study’s phenomenological orientation, acknowledging that meaning is coconstructed through participants’ accounts and researchers’ interpretive engagement.

Data from the focus groups and the teacher interview were analyzed using a hybrid abductive analytic approach that combined sensitizing concepts derived from the TPACK framework (technology, pedagogy, and content) and the analytical axes of the RAÍCES project (motivation, facilitators, barriers, perceived learning, and transfer), together with an inductive, reflexive thematic analysis following Braun and Clarke [7]. Rather than functioning as a closed coding template, these theoretical dimensions served as an initial analytical scaffold to support familiarization with the data and the preliminary organization of participants’ accounts. This method was selected for its theoretical flexibility and its compatibility with interpretive qualitative approaches, allowing for the identification of patterns of shared meaning while preserving sensitivity to participants’ lived experiences. This process enabled deeper analysis and the integration of both theoretical categories and meanings constructed from participants’ lived experiences.

Throughout the analytic process, the analysis followed a hybrid abductive approach in which theoretical concepts and data-driven insights informed one another iteratively. The TPACK dimensions and the RAÍCES analytical axes were used as an initial organizing structure, a sensitizing framework that provided a preliminary scaffold for indexing participants’ accounts, rather than as a closed coding scheme. Building on this structure, inductive open coding was conducted to capture meanings not anticipated by the framework, such as the salience of the visual component and the emotional ambivalence of competition. Theme construction was therefore reflexive and primarily inductive: deductively derived categories were retained only when supported by the data, and emergent codes that exceeded the framework were preserved and developed into themes in their own right. Throughout this process, the research team moved iteratively between the empirical material and the theoretical framework, so that the final themes reflect participants’ lived experiences while remaining interpretable through the TPACK lens. The full analytical workflow is summarized in Multimedia Appendix 2, which illustrates the iterative integration of deductive template coding and inductive reflexive thematic analysis, showing how theoretical categories served as an initial analytic scaffold while allowing themes to emerge from the data.

This hybrid analytic process generated the 6 thematic outcomes described in the *Results* section: “Techno-Disciplinary Alignment and Visuality,” “Kahoot! as a Formative Closure: Rapid Check, Review, and Gap Diagnosis,” “From Score to Learning: Feedback as a Condition for Being Truly Formative,” “Motivation and socioemotional climate: between play, competition, and psychological safety,” “Conditions of implementation: facilitators and barriers situated in the course context,” and “Projection and improvement: from a single activity to a sustainable and transferable strategy.”

The analysis was conducted collaboratively by members of the research team. The ATLAS.ti (Lumivero, LLC) software was used as a support tool for the management, organization, and coding of qualitative data.

### Ethical Considerations

The study was approved by the institutional scientific ethics committee (CEC; protocol number 40‐25, approved on June 25, 2025). Participation was voluntary, and all participants signed an informed consent form prior to data collection. Participants were also informed that they could withdraw from the study at any time, without any academic or other consequences.

The collected data were accessible exclusively to the research team and were stored in private folders within the institution’s secure cloud storage system (OneDrive). To ensure confidentiality, participants’ names were replaced with pseudonyms in transcripts and in the presentation of results, with identifying information kept under restricted access.

### Researcher Positionality and Reflexivity

To ensure qualitative rigor, the research team engaged in continuous reflexivity throughout the study design, data collection, and analysis. The primary analysis was conducted by 4 fourth-year medical students (SPAF, EVZ, VFRV, and RVCA) and supervised by 2 faculty physicians (AHA and CRB), all of whom were actively involved in the RAÍCES 2 educational project. The student researchers shared a common institutional and academic background with the participants, having previously completed the same morphology I course at Universidad San Sebastián. This insider positionality facilitated a deep, empathetic understanding of the students’ lived experiences and the specific pressures of practical assessments. However, it also introduced potential biases about preconceived notions of student anxiety and course difficulty.

To manage these subjectivities and maintain an interpretive balance, reflexivity was operationalized through a collaborative, iterative coding process. The student researchers held weekly peer-debriefing meetings to compare preliminary codes generated in ATLAS.ti and discuss their interpretive lenses.

When disagreements emerged regarding the boundary or meaning of specific codes or subthemes, they were not resolved by a majority vote; instead, they were critically debated and cross-examined alongside the raw transcripts and under the guidance of the faculty members until an analytical consensus was reached. Alternative interpretations of participants’ accounts were deliberately explored during these discussions, and emerging themes were repeatedly challenged against the full dataset to ensure that interpretations remained grounded in the data rather than reflecting researchers’ initial assumptions or preferred explanations. Themes were iteratively refined through multiple rounds of dialogue, moving back and forth between the empirical data and the TPACK theoretical framework to ensure that the final interpretations truly reflected the participants’ accounts rather than the researchers’ preexisting assumptions.

## Results

### Theme 1: Techno-Disciplinary Visual Alignment

Kahoot!’s use was sustained by its intuitive interface and its direct alignment with the visual demands of the course. Participants valued the tool when it effectively simulated the formal objective structured practical examinations, such as gymkhanas (a local term for timed, station-based practical assessments analogous to objective structured practical examinations) by prioritizing the visual recognition of anatomical structures under time pressure. Quick entry via QR codes facilitated a seamless start without disrupting the laboratory flow.

Furthermore, although the image-based pin function required a brief initial learning curve regarding pointer manipulation, it demonstrated high formative potential for anatomical tasks. Ultimately, both students and the teacher emphasized that the success of the activity depended critically on using authentic, high-resolution images identical to physical laboratory resources (eg, physical models and anatomical tables) to effectively train the clinical eye.

### Theme 2: Kahoot! as a Formative Closing Tool

Implemented as an end-of-class activity, Kahoot! effectively structured the laboratory session’s closure. Acting as an integrative “exit ticket,” the platform provided a crucial opportunity for immediate self-assessment. It allowed students to immediately apply the reviewed concepts and contrast their subjective perceived understanding with their objective performance.

Rather than introducing new material, both students and the teacher valued the tool primarily as a diagnostic resource to identify learning gaps and estimate the achievement of expected outcomes. Consequently, this rapid formative check helped guide subsequent independent study. However, its impact on long-term study habits varied; while some students integrated the platform into their personal study routines to simplify content, others viewed it strictly as a classroom complement that did not alter their established study methods.

### Theme 3: The Role of Immediate Explanatory Feedback

While Kahoot!’s playful elements such as speed and ranking initially engaged students, these gamified features alone were insufficient for meaningful learning. The tool’s true formative value depended entirely on the teacher’s pedagogical mediation. Participants stressed that merely identifying correct or incorrect answers was inadequate; explicit explanatory feedback was required to understand the rationale behind the errors. Crucially, both students and the teacher noted that this feedback needed to be immediate, leveraging the brief interval between questions while the visual stimulus remained active in students’ working memory.

However, this created a persistent pedagogical tension: teachers had to constantly balance the game’s dynamic rhythm with the necessity of pausing for deeper conceptual discussions, a task that proved particularly challenging with complex or extensive content.

Furthermore, the effectiveness of this feedback loop was heavily reliant on precise question design. Learning was notably hindered when images lacked clarity or when items used terminology that deviated from the course’s standard anatomical vocabulary.

### Theme 4: Socioemotional Dynamics of Gamification

Gamification effectively disrupted the standard classroom routine, adding dynamism and recapturing sustained students’ attention. While competition served as a powerful motivational driver for personal improvement, it also generated emotional ambivalence. Students experienced initial evaluative nervousness, and participants recognized a risk of demotivation or frustration for those consistently achieving lower rankings. In this context, the platform’s anonymity was identified as a crucial safeguard. It fostered psychological safety by allowing students to participate and take academic risks without the fear of public failure.

### Theme 5: Contextual and Logistical Implementation Factors

The successful implementation of Kahoot! was heavily mediated by contextual and logistical variables. Functioning effectively as an end-of-class closure activity (10‐15 min), it helped recapture waning attention without disrupting laboratory dynamics. However, students faced minor technological barriers, such as device-battery anxiety and reliance on institutional Wi-Fi, raising concerns about digital equity and exclusion. While the teacher found the platform intuitive to facilitate, students recognized the substantial workload required to design high-quality visual quizzes. Isolated technical and terminological incidents occurred but were quickly resolved without detracting from the overall positive evaluation.

### Theme 6: Projections for Sustainable Pedagogical Use

Looking forward, participants projected that systematizing the use of Kahoot!, rather than using it as an isolated intervention, would yield a deeper impact on consistent study habits. To ensure sustainability and alleviate individual faculty workloads, students recommended developing a standardized, institutionally supported question bank. This repository should ensure disciplinary balance, specifically by integrating more histology items alongside macroscopic anatomy. Ultimately, participants affirmed that the core competencies practiced through this gamified tool, such as rapid image recognition and decision-making under time constraints, were directly transferable to formal practical assessments.

Reflexive thematic analysis identified 6 main themes that synthesize students’ and the teacher’s perceptions of the use of Kahoot! as a formative assessment strategy in morphology I. These themes reflect how the educational value of the tool emerged from the interaction between the visual and temporal design of the activities, the teacher’s pedagogical mediation, the socioemotional climate generated by gamification, and contextual implementation conditions. Table 1 presents an interpretive synthesis of the identified themes. To preserve the inductive depth of the participants’ accounts, these findings are presented chronologically based on their lived experiences, using the TPACK framework strictly as a subsequent interpretive lens in the discussion section.

**Table 1.** Main themes identified from the reflexive thematic analysis of the use of Kahoot! in morphology I.

Theme	Core meaning
Alignment with disciplinary and visual demands	Kahoot! was perceived as valuable when its visual design, task structure, and available time reproduced conditions similar to practical assessments and laboratory work.
Kahoot! as a formative closing activity	The activity functioned as a rapid formative check at the end of class, supporting self-assessment, identification of learning gaps, and guidance for subsequent study.
From score to learning: feedback as a critical condition	The formative value of the tool depended on immediate explanatory feedback from the teacher, which transformed game results into opportunities for meaningful learning.
Motivation and socioemotional climate	Gamification increased engagement and attention but also generated ambivalent emotional responses related to competition, time pressure, and performance visibility.
Contextual conditions of implementation	Factors such as connectivity, device availability, class time management, and teacher preparation conditioned the quality of the experience.
Projection toward sustainable use	Participants envisioned greater educational impact through more systematic use, improved item design, disciplinary balance, and institutional support.

## Discussion

### Principal Findings and Interpretation

In summary, the main findings of this study indicate that Kahoot! can effectively function as a formative assessment tool when its visual and temporal design aligns with authentic practical examinations. Furthermore, as experienced by the participants, its educational value is not inherent in the technology itself but relies crucially on the participating instructor’s immediate explanatory feedback to mitigate competitive anxiety and transform gamified scores into meaningful learning.

The primary perception of Kahoot! as a low-stakes formative environment, rather than a summative measurement, highlights a successful pedagogical integration of technology to alleviate evaluative anxiety. From the perspective of the TPACK framework, this shift illustrates the pedagogical use of technology to support formative assessment, demonstrating how a digital tool technological knowledge (TK) can be intentionally steered away from evaluative pressure to serve a purely diagnostic purpose pedagogical knowledge (PK) within a demanding discipline content knowledge (CK). By leveraging technological affordances specifically for formative closure, the classroom dynamic transitions from performance-oriented competition to a psychologically safe learning space where errors are embraced as pedagogical milestones for gap diagnosis.

Crucially, Kahoot!’s pedagogical value depended entirely on immediate explanatory feedback. Students found scores and rankings insufficient without clear justifications for correct and incorrect answers. In this sense, learning did not occur during the act of responding to the question but rather during the guided reflection that followed it, positioning the teacher’s mediation as a central component perceived to support the transformation of errors into meaningful learning opportunities.

This friction between engagement and cognitive processing illuminates the critical pedagogical and socioemotional dimensions of technology use in high-stakes environments. Viewed through the lens of the TPACK framework, this finding reveals that when the inherent characteristics of a technology (TK), such as Kahoot!’s speed and countdown timer, override the needs of the content (CK), a direct tension arises with the pedagogical objectives (PK). In disciplines with a high cognitive and visual load, like morphology, technological fluency can conflict with the time required for conceptual consolidation.

This correlation between item design flaws and student errors underscores the critical need for a tight integration of disciplinary content, pedagogical decisions, and technological affordances. Viewed through the lens of the TPACK framework, this issue illustrates what happens when the technological execution (TK) fails to precisely mirror the unique visual demands of the content (CK). In a highly visual discipline like morphology, where anatomical pattern recognition is a core competency, the graphical fidelity of a digital tool is not a mere esthetic preference; it is a pedagogical prerequisite. When images lack clarity or distractors introduce terminological variance, the technological tool (TK) creates a barrier that interferes with the pedagogical goal (PK) of accurate self-assessment.

The coexistence of enthusiasm and evaluative anxiety further illuminates the pedagogical and socioemotional dimensions of technology use, revealing that gamified environments activate learners at both cognitive and affective levels. Viewed through the TPACK framework, the emotional impact of a digital tool (TK) is not neutral; rather, it is heavily modulated by how game mechanics (such as leaderboards and speed points) intersect with the pedagogical climate (PK) designed by the instructor. While competition serves as a powerful driver for engagement, persistent low rankings introduce socioemotional friction in which continuous peer comparison risks shifting student anxiety from a state of productive alertness to frustration and demotivation. In the face of these adverse situations, strategies that foster student security become paramount (such as real-time reassurance provided by the instructor and student anonymity), thereby ensuring that the competitive drive does not compromise psychological safety. Specifically, anonymity acts as a structural prerequisite embedded within the technological interface (TK) that mitigates the fear of public exposure, promoting broader participation by allowing students to take cognitive risks even when unsure of their answers. Without these supportive, feedback-oriented instances (PK) to buffer the pressures of the game, the technological tool (TK) loses its formative value and defaults to an evaluative stressor. Indeed, this anonymity facilitates active participation without fear of public exposure, reducing the dread of making mistakes, whereas competition remains an ambivalent strategy that requires careful pedagogical scaffolding to prevent demotivation when peer comparison becomes persistent.

The tight interdependence among these emergent findings demonstrates that a digital gamified environment alone remains pedagogically inert without deliberate technological, pedagogical, and content alignment. This systemic interaction offers a clear projection toward sustainable use and provides transferable design principles for educators in other visually intensive medical disciplines, such as histology, pathology, or radiology. To successfully transfer these insights, instructors must move away from using gamified tools as isolated recreational games. Instead, sustainable implementation requires a balanced articulation within the TPACK framework: first, ensuring that the technological execution (TK) achieves authentic visual alignment with disciplinary content demands (CK); second, positioning immediate, explicit teacher feedback (PK) as a strict prerequisite to automated scoring; and third, actively managing the socioemotional climate to preserve psychological safety. Rather than a mere classroom intervention, these conditions establish a reproducible pedagogical model that transforms gamified formative assessment into a sustainable tool for diagnostic gap recognition across medical curricula.

### Comparison With Prior Work

The findings of this study are consistent with the literature indicating that the educational value of digital technologies in medical education depends less on the tool itself and more on its integration with the pedagogical and disciplinary demands of the learning context. In their review of the digital teaching landscape in medicine, Yeung et al [1] describe that technological interventions tend to be better valued when they are coherently aligned with learning objectives and with the practices inherent to each course. In line with this, our findings show that Kahoot! was perceived as useful mainly when it aligned with the practical performance required in morphology I, particularly the recognition of anatomical structures through authentic images under time constraints comparable to the course’s practical assessments.

Previous studies have documented that the use of quiz-based and game-based platforms can foster engagement and content review among medical students. Both Yuenyongviwat and Bvonpanttarananon [2] and Cordero-Cordero et al [4] report positive perceptions of Kahoot! as a learning support strategy. However, the results of the present study add an important nuance by showing that a playful and competitive component alone does not guarantee learning perceived as meaningful. In this case, explanatory feedback provided by the teacher emerged as the central condition that transformed the game dynamics into a genuinely formative experience, in line with what Rodríguez Salvador and Arboniés Ortiz [8] propose regarding the role of feedback in formative assessment in medical education.

From a motivational standpoint, the results partially align with studies describing gamification as a strategy capable of increasing student participation and interest. Rivera et al [3] note that technology-mediated interactive activities can foster engagement in medical teaching contexts. However, the present study offers a more complex view by evidencing that motivation associated with Kahoot! is highly ambivalent. While competition was described as stimulating for many students, accounts of evaluative anxiety and potential demotivation also emerged when peer comparison became persistent. This observation is consistent with findings by Kirsch and Spreckelsen [9], who warn that competitive gamification may generate initial enjoyment but not necessarily sustained benefits for intrinsic motivation or learning.

Crucially, this socioemotional vulnerability underscores broader digital health risks documented in recent literature. As demonstrated by Song et al [10] in their evaluation of classroom technology, inappropriate or excessive use of unintegrated digital tools can be positively correlated with academic burnout among university students. To mitigate these psychological risks, Song et al [10] emphasize the importance of enhancing teacher leadership in the digital classroom and strengthening student resilience. This directly supports our findings, suggesting that when a tool like Kahoot! is used under high time and visual constraints, the emotional toll can easily tip toward burnout or frustration if the tool functions in isolation. Therefore, robust pedagogical design and active instructor mediation are required to safeguard the socioemotional climate.

Taken together, these integrated findings strongly reinforce the propositions of the TPACK framework by Mishra and Koehler [5,6], showing that the effectiveness of educational technologies in medical training does not lie, in the technological component in isolation but in the deliberate interaction between disciplinary knowledge (content), pedagogical decisions, and the intentional use of technology. From this perspective, the present study contributes situated qualitative evidence that deepens the understanding of how gamified tools such as Kahoot! are experienced by students and teachers when integrated into practical medical contexts characterized by high cognitive, evaluative, and socioemotional demands.

### Conceptual Contribution of the Study

Rather than demonstrating the effectiveness of a specific digital platform, this study identifies a set of pedagogical conditions that appear to be essential for designing effective gamified formative assessment in visually intensive medical courses. These principles were inductively derived from 6 themes identified in the reflexive thematic analysis and subsequently interpreted using the TPACK framework.

First, gamified activities should reproduce the authentic visual and temporal demands of disciplinary assessments to ensure alignment with learning objectives. Second, game mechanics should be embedded within intentional pedagogical design rather than functioning as isolated motivational elements. Third, immediate explanatory feedback should accompany each response to transform game performance into meaningful learning. Finally, implementation should explicitly consider students’ socioemotional experiences by balancing engagement, competition, and psychological safety.

Together, these findings extend the application of the TPACK framework by proposing transferable design principles that can inform the development of gamified formative assessment beyond the specific context of morphology I and the use of Kahoot!

### Strengths and Limitations

This study is distinguished by a comprehensive qualitative approach that enabled a rich and detailed understanding of students’ and the teacher’s experiences regarding the use of Kahoot! in morphology I. Methodological triangulation, integrating 2 student focus groups and 1 semistructured interview with the teacher, strengthened the credibility of the findings by contrasting pedagogical intentions with students’ perceptions and needs.

The combination of a deductive theoretical framework based on the TPACK model and the RAÍCES project axes with an inductive reflexive thematic analysis (Braun and Clarke [7]) allowed for a systematic and nuanced interpretation of the data, identifying both expected patterns and relevant emergent elements, such as the importance of the visual component and the ambivalence of competition.

Among the limitations, the study was conducted in a single institutional context, the Universidad San Sebastián Valdivia campus, and with a specific cohort of medical students, which limits the transferability of the findings to other settings. Additionally, it is necessary to acknowledge that the faculty perspective is derived from a single instructor, meaning these specific pedagogical reflections may not represent the broader teaching body of the institution. Crucially, because this research was based on a single Kahoot! intervention, a potential “novelty effect” must be critically considered. The high levels of student motivation, focus, and even the acute evaluative anxiety reported may have been heavily influenced by the unique, disruptive nature of the activity within the standard laboratory routine. If the tool were integrated into a repeated or mandatory framework, these dynamics would likely shift; repeated exposure might normalize the format, thereby mitigating acute time-related anxiety, yet it concurrently risks inducing technological fatigue and diminishing the situational interest observed in this isolated trial.

Furthermore, our recruitment relied on a voluntary sampling strategy, which introduces a distinct self-selection bias. It is highly probable that the 14 students who chose to participate represented more academically engaged or more positive voices within the cohort, potentially overrepresenting favorable perceptions of Kahoot! Consequently, the perspectives of students who experienced severe demotivation, lower academic performance, or technological alienation may have been underrepresented, meaning the full spectrum of socioemotional friction was not entirely captured. Finally, the qualitative approach did not incorporate objective measures of academic performance, and the implementation under a bring your own device model introduced variability in devices and connectivity, which heavily conditioned the individual user experience.

### Future Directions

Future studies could evaluate the impact of a systematic and longitudinal implementation of Kahoot! as a formative assessment strategy in morphology I on objective academic performance indicators, using quasi-experimental designs or comparisons between course sections. It would also be valuable to explore, in a controlled manner, the effects of design variables identified as critical in this study—such as the quality of visual materials, the use of timed responses, and the presence of explanatory feedback—while incorporating complementary measures such as motivation, self-efficacy, cognitive load, and assessment-related anxiety.

### Conclusions

#### Principal Insights and Disciplinary Transferability

This study explored the experiences of students and a teacher regarding the use of Kahoot! as a formative assessment strategy in the morphology I laboratory, showing that its pedagogical value does not lie in the technological tool itself, but rather in its alignment with the disciplinary, evaluative, and pedagogical demands of the course. Kahoot! was perceived as useful when it allowed students to practice the recognition of structures using authentic images under time constraints and in formats comparable to real practical assessments.

In this context, Kahoot! can constitute a relevant and transferable strategy in health sciences education, provided that it is implemented intentionally, with well-designed items, explicit pedagogical feedback, and a conscious consideration of its cognitive and emotional implications. Conceptually, our findings regarding the interplay between rapid image recognition and real-time faculty mediation possess significant transferability to other visually intensive medical disciplines, such as histology, pathology, and radiology. In these fields, undergraduate students face a steep learning curve characterized by a high visual cognitive load, in which they must transition from merely viewing complex structures to instantly recognizing clinical patterns under diagnostic time constraints. By demonstrating that gamified environments can effectively simulate these authentic evaluative pressures, our study offers a broader pedagogical model: digital tools serve as a valuable sandbox to test the “clinical eye,” but their educational efficacy remains largely dependent on immediate instructor deconstruction of visual errors to prevent cognitive overload and safely foster diagnostic competence.

The findings indicate that within the scope of the analyzed experiences, the tool functioned primarily as a formative closing activity, supporting self-assessment, diagnosis of learning gaps, and consolidation of learning, rather than as a summative measurement mechanism. However, its formative impact was critically conditioned by the participating instructor’s mediation, particularly through the provision of explanatory feedback following each question.

From a socioemotional perspective, gamification fostered motivation, participation, and attention but also activated ambivalent emotional responses associated with competition and time management. These results underscore the need for careful pedagogical design that considers not only students’ engagement but also learners’ psychological safety.

#### TPACK-Informed Design Principles and Practical Recommendations

Taken together, the results support the TPACK framework by suggesting that the perceived use and educational value of gamified tools in medical education emerge from the interaction between technology, pedagogy, and disciplinary content. More broadly, these findings suggest that the effectiveness of gamified formative assessment depends less on the digital platform itself than on the pedagogical conditions under which it is implemented. Authentic alignment with disciplinary tasks, immediate explanatory feedback, intentional instructor mediation, and attention to students’ socioemotional experiences emerged as potentially transferable design principles that may guide the implementation of gamified formative assessment across visually intensive health professions education.

These findings suggest that educators implementing gamified formative assessment should approach these tools not as stand-alone games but as highly intentional pedagogical interventions. Instructors in visually demanding disciplines should specifically design digital quizzes to mirror the authentic time constraints and visual fidelity of real-world practical assessments. Furthermore, rather than relying on the platform’s automated scoring, educators must actively deliver immediate, explanatory feedback between questions. This real-time mediation is essential to transforming the inherent competitive anxiety of gamification into a psychologically safe and meaningful learning opportunity.

## Supplementary material

10.2196/90651 Multimedia Appendix 1 Focus group and semistructured interview guides.

10.2196/90651 Multimedia Appendix 2 Methodological flow diagram of the qualitative data analysis process.

## Abbreviations

CK: content knowledge
PK: pedagogical knowledge
RAÍCES 2: Red de ayudantes de investigación comprometidos con la educación en salud (Network of Student Researchers Committed to Health Professions Education)
TK: technological knowledge
TPACK: Technological Pedagogical Content Knowledge
