# Supplementary material for: Gamified Formative Assessment in a First-Year Medical Morphology Course: Qualitative Study
Source: JMIR Hum Factors. 2026 Oct 22;13:e90651. doi: 10.2196/90651 (PMC13717265; doi:10.2196/90651)
Supplement: Supplementary file 1 [file humanfactors-v13-e90651-s002.pdf]

HYBRID (ABDUCTIVE) THEMATIC ANALYSIS WORKFLOW
INTEGRATION OF SENSITIZING THEORETICAL CONCEPTS (TPACK AND RAÍCES) WITH INDUCTIVE REFLEXIVE THEMATIC ANALYSIS

1. Familiarization

Verbatim transcription of the two focus groups and the teacher interview; repeated reading and immersion in the data.

2. Initial coding using sensitizing concepts (TPACK and RAÍCES)

TPACK dimensions and RAÍCES analytical axes served as sensitizing concepts to support familiarization with the data and the preliminary organization of participants' accounts, rather than as a fixed coding template.

TPACK dimensions: Technology • Pedagogy • Content
RAÍCES analytical axes: Motivation • Facilitators • Barriers • Perceived learning • Transfer
Used to organize participants' accounts, not to constrain theme development.

3. Inductive open coding (data-driven)

Data-driven coding captured meanings not anticipated by the theoretical framework, such as the salience of visual recognition and the emotional ambivalence associated with competition.

Emergent codes preserved and developed in their own right.

4.Integration: Candidate theme construction

Sensitizing concepts and inductively generated codes were iteratively integrated into candidate themes.
Theoretical concepts were retained only when supported by the data, while emergent codes extending beyond the framework developed into new themes.

5. Iterative review and refinement

Repeated movement between the transcripts and the theoretical framework through collaborative analytic discussions; coding managed in ATLAS.ti..

Themes repeatedly challenged against the complete dataset and refined across multiple analytic rounds

6.Defining and naming themes

Six final interrelated themes conceptually interpretable through the TPACK framework.
Representative quotations selected.

CODING LOGIC

- **Sensitizing theoretical concepts:** TPACK dimensions and RAÍCES analytical axes used to orient and organize the analysis.
- **Inductive:** Data-driven meanings emerging from participants' accounts.
- **Reflexive integration and theme development:** Iterative integration of theory and data to construct and refine themes.
- - - - Iterative loop (back-and-forth throughout analysis)

Analysis followed Braun and Clarke's reflexive thematic analysis within a hybrid abductive analytic approach, using TPACK and the RAÍCES analytical axes as sensitizing concepts while allowing themes to emerge inductively through iterative engagement with the data.
