# Supplementary material for: Gamified Formative Assessment in a First-Year Medical Morphology Course: Qualitative Study
Source: JMIR Hum Factors. 2026 Oct 22;13:e90651. doi: 10.2196/90651 (PMC13717265; doi:10.2196/90651)
Supplement: Supplementary file 2 [file humanfactors-v13-e90651-s001.docx]

# Multimedia Appendix 1

## TRANSCRIPCION FOCUS GROUP 1 (29 de septiembre 2025)

**Moderador**: Hola, ¿me escuchan? Voy a encender la cámara. Hola a todos; ya me tuvieron que haber visto el otro día, pues estaba junto a Sofía en la firma de los consentimientos informados. Soy Eduardo y voy a estar a cargo del “focus group” de hoy. Eso sí, faltan personas y la idea es que estemos todos. Participante 4, Participante 3, ¿saben algo de ellos? Me parece que son quienes faltan.

**Participante** **1**: Participante 4 está intentando unirse.

**Moderador**: Ah, muy bien; esperaremos un momento más. Oficialmente, me parece que ya estamos todos. Bueno, ¿por dónde comienzo? Me presento de nuevo: soy Eduardo y estuve el otro día en la firma del consentimiento informado. Voy a estar a cargo de este “focus group”. En dicho consentimiento ya les tuvieron que haber explicado la dinámica de esta reunión. Al final, yo plantearé unas preguntas, por así decirlo. Ustedes respondan y siéntanse libres de opinar y contar cómo fue su experiencia con la tecnología que estuvieron ocupando. Bueno, me parece que la grabación ya está en curso. Es importante que quede registrado que todos ustedes están dando su consentimiento para ser grabados. Les voy a pedir que, por favor, abran sus micrófonos y digan algo como: "Hola, sí, estoy de acuerdo con ser parte de la grabación". Participante 2, ¿todo bien con tu micrófono?

**Participante 2**: Ahora sí, ahora sí. Muchas gracias.

**Moderador:** Qué bueno. Justo estaba diciendo que es importante que en la grabación queden sus consentimientos registrados. ¿Están todos de acuerdo con ser parte de la grabación para el “focus group”?

**Participantes**: Hola, sí.

**Moderador**: Vi que todos sus micrófonos se encendieron, así que voy a confiar. Para comenzar, el primer tópico del que tenemos que hablar es su experiencia al inicio del desarrollo de esta tecnología. Nos interesaría saber un poco acerca de: cuando piensan en la clase a la cual se les aplicó el Kahoot, ¿qué es lo primero que recuerdan de esa experiencia? Primero tómense un tiempo para pensar, no se preocupen.

**Participante 2**: Al menos por mi parte, un poco de nerviosismo porque llegó de imprevisto, pero fue entretenido.

**Moderador:** Ah, ya. ¿Podrías explicar un poco más, si es que quieres, por qué sentiste esos nervios? ¿Qué tenía de diferente esta clase?

**Participante 2**: Es que, como fue un poco de sorpresa, no venía mentalmente preparado para ser evaluado; pero siento que, de todas formas, el hecho de que haya sido inesperado incentivó a que nos preparáramos para el momento exacto.

**Moderador**: Ya. ¿Alguien comparte el pensamiento de participante 2 o no está de acuerdo?

**Participante 4**: Yo, por lo menos, tuve emociones diferentes a las de participante 2, porque según yo fue una actividad súper relajada. El profesor nos explicó de qué iba a tratar, aclaró que obviamente no era evaluado ni nada, y la encontré súper dinámica y entretenida. Estar todo el rato pendientes y atentos a la clase nos ayudó un poco a relajarnos y, al final, consistía en aplicar lo que estábamos viendo.

**Moderador**: ¿Entonces, en general, la experiencia les genera recuerdos positivos?

**Participante 4**: Por lo menos a mí, sí.

**Participante 2:** Por mi lado también. Quizá sonó un poco como si hubiera sido negativo, pero no.

**Participante 5**: (...)

**Moderador**: ¿Podrías, por favor, repetir lo que dijiste?

**Participante 5**: Sí, estaba esperando a que se me dejara de pegar la conexión. Iba a decir que lo sentí como una emoción, porque como tengo buena experiencia con Kahoot y los encuentro entretenidos, dije "¡guau!". Fue como "¡ah, qué bacán!".

**Moderador**: Creo que tengo preguntas más adelante que van a explorar eso, pero ¿habían ocupado Kahoot antes? ¿Conocían Kahoot?

**Participante 5:** Sí, varias veces en el colegio y cosas así.

**Moderador**: Ya, qué bueno. Entonces ya conocían esta herramienta, no fue una sorpresa total.

**Participante 5**: Sí, sabía que había que responder bien rápido y toda la cosa.

**Moderador:** Ya. Y por ejemplo, pasando a otro tópico, ¿cómo describirían su primera impresión al usar estas tecnologías en las asignaturas clínicas? Va muy relacionado con lo que recién estábamos hablando, pero piensen en una palabra o algo que se les venga a la mente.

**Participante 3:** Bueno, yo quiero aportar en relación con lo anterior y a la pregunta que hiciste. Yo lo encuentro súper divertido, en realidad, porque siento que, al ser de sorpresa, fue un poco chocante o desafiante el saber qué tanto sabías. También nos sirvió porque después teníamos una evaluación de eso; entonces, de cierta forma, nos hizo preocuparnos y darnos cuenta de si estábamos preparados para esa evaluación. Siento que sirvió mucho, ya sea para la evaluación o para saber qué tanto sabíamos de lo que habíamos aprendido hasta ese momento.

**Moderador**: Qué bueno. Conque dices que les causa una impresión fuerte, los mantiene alerta y preparados para futuras evaluaciones.

**Participante 1:** Sí, para mí igual fue de sorpresa, pero lo encontré bueno porque era mucho más dinámico. Y, como dijo mi compañera, así podía saber en qué nivel estaba y enfocarme en lo que noté que estaba fallando.

**Moderador:** Qué bueno que digan eso y que les haya gustado. Entonces, ¿consideran que les sirvió bastante el Kahoot?

**Participante 4**: Sí, totalmente.

**Participante 5**: Yo creo que sirvió, pero también había partes que no tenían tanta relación con lo que habíamos pasado. Había preguntas de hormonas y cosas así que no vimos directamente, sino que uno las sabía, no sé, por la PAES o por el colegio, pero no eran directamente temas de anatomía que hubiéramos revisado. Entonces, las preguntas que eran más de identificar estructuras siento que sirvieron porque permitían aplicar lo de la clase; las otras eran muy generales.

**Participante 1:** Yo igual creo que sirvió, porque así en el control ya teníamos la estructura de cómo iba a ser, ya que no todos hemos dado antes un control de morfología y así no íbamos a estar tan perdidos. Con respecto al tema de las preguntas, claro, había algunas de estructura y otras de saber qué hormona era, pero eso igual nos sirve para profundizar más en el tema y no quedarnos solo en la estructura.

**Moderador:** Qué bueno; entonces tuvo algunas dificultades para alinearse con los conocimientos específicos que estaban viendo, pero aun así fue pertinente. La última pregunta del inicio va relacionada con qué expectativas tenían antes de utilizar las tecnologías y qué tan distintas fueron en la realidad al aplicar el Kahoot.

**Participante 4:** ¿Podrías repetir la pregunta, por favor?

**Moderador:** Sí, se refiere a las expectativas que tenían antes de utilizar el Kahoot y qué tan distintas fueron en la realidad. Creo que había escuchado a alguien, pero no identifiqué quién encendió el micrófono.

**Participante 5:** Yo iba a decir que tenía expectativas bajas, pensaba: "me va a ir mal, no voy a saber nada". A pesar de que recién habíamos aprendido las cosas de anatomía, pensé que iba a perder o que iba a estar adivinando, presionando lo primero que se me ocurriera. Pero al final no fue así; sirvió porque pude aplicar de inmediato lo que habíamos aprendido. Por ejemplo, hicieron varias preguntas de estructuras que recién habíamos visto y fue bueno, porque aplicar al tiro lo explicado me hizo recordar qué había dicho el profesor para responder.

**Moderador:** Ya, aplicando de inmediato el conocimiento de la clase teórica y del laboratorio.

**Participante 5:** Sí.

**Moderador:** Qué bueno que pueda servir para integrar. ¿Alguien más siente que el uso del Kahoot superó sus expectativas?

**Participante 3**: A mí sí, porque iba con el tiempo en contra y también obtenías mayor puntaje al responder antes en comparación con tus compañeros. Igual te acercaba más a la realidad de las evaluaciones que tenemos, como los controles o las gincanas (que todavía no tenemos ninguna, pero también son con tiempo). Entonces, te das cuenta de cómo reaccionas tú, si te pones nervioso o si trabajas bien bajo presión. Al principio tenía un poco de miedo por ser el primer acercamiento, pero después superó las expectativas; se puede sobrellevar y aprender a trabajar con ese tiempo, que es como un minuto y medio o dos minutos, un poco más que en el Kahoot.

**Moderador:** Ya, entonces los va adecuando al estrés que se siente en las pruebas y al realizar las evaluaciones prácticas. ¿Y alguien de acá tenía más expectativas antes y estas disminuyeron con el Kahoot? Pueden ser completamente honestos, se me olvidó decir eso. No necesito que me digan que todo está bien; pueden decir si estuvo mal o si les hubiera gustado que fuera mejor.

**Participante 4**: A mí me hubiese gustado lo que dijo participante 5: que el Kahoot estuviera enfocado tal vez solamente en laboratorio y, eventualmente, hacer otro Kahoot netamente teórico, para poder haberlo diferenciado.

**Participante 3**: Sí, y también agregando lo que dijo participante 4, si mal no recuerdo, creo que no había o había muy pocas preguntas de lo que era histología; el Kahoot iba muy ambientado a anatomía. Como morfología abarca ambos ramos, pudieron haber incluido más preguntas enfocadas en el laboratorio de histología y anatomía.

**Participante 5:** Es verdad, eso no lo había pensado. No había ninguna pregunta de eso y, para reforzar, igual falta esa parte.

**Moderador:** Ya, entonces sugieren una integración de la anatomía macroscópica, microscópica y también más enfocado en lo práctico. ¿Alguien más que le gustaría añadir algo? ¿Nadie? Antes de que continuemos con la siguiente pregunta, se me olvidó mencionar que no es obligatorio, pero si desean encender sus cámaras sería mejor para la reunión. Pasando a las preguntas que abarcan la dimensión tecnológica, les quería preguntar: ¿qué obstáculos técnicos enfrentaron y cómo los resolvieron durante la actividad? ¿Hubo siquiera algún obstáculo?

**Participante 5**: Yo personalmente creí que no iba a poder hacer el Kahoot porque tenía poca batería; estaba nerviosa y creía que se me iba a apagar el teléfono, tenía como 3% y dije: "no lo voy a poder hacer". Además, tengo el teléfono en otro idioma y pensé que me iban a salir todas las preguntas en ese idioma, pero después me di cuenta de que se podía cambiar el idioma solo en el Kahoot. Fueron más cosas de mi celular que del Kahoot en sí.

**Moderador**: Claro, esa es otra variable: el problema de la herramienta es que depende de los dispositivos de cada alumno, no es algo que entregue la universidad, así que es súper válido. ¿Alguien más tuvo algún problema?

**Participante 5**: Se demora en cargar a veces.

**Participante 3**: Al menos en mi sección, lo que pasó fue que el Kahoot traía incluidas a personas de otra sede o de otra sección, no sé bien. Hicimos un intento con las dos primeras preguntas y empezaron a salir con mayor puntaje personas que no estaban en la sección. Ahí el profesor tuvo que buscar cómo borrar todo eso para que estuviéramos presentes solo los que nos encontrábamos en ese momento y no quedaran guardados los datos de la sección anterior o de otra sede.

**Moderador**: Oye, qué raro. ¿Pero lo pudieron solucionar al final?

**Participante 3**: Sí, el profesor buscó la opción donde se podía borrar todo el historial de las personas inscritas anteriormente. Por ejemplo, salía que "Juanito Pérez" era el número uno, pero no había ningún Juanito Pérez en esa sección; quedaban guardados los porcentajes de gente que ya había hecho el Kahoot antes. Como mi sección es una de las primeras, yo creo que después no le pasó lo mismo al profesor porque ya sabía cómo eliminarlo.

**Moderador**: Ah, ya, qué bueno. Qué bien que se solucionara ese problema. ¿En alguna otra sección se presentó algo parecido o algún otro obstáculo?

**Participante 4:** No, en la mía nada.

**Moderador:** Qué bueno. La siguiente pregunta se relaciona con: ¿Qué funciones les resultaron especialmente útiles y cuáles consideran que tuvieron un impacto limitado? ¿Alguien?

**Participante 5**: La función del pin. Yo no sabía que se podía hacer eso de poner un pin en la estructura, pero tenía una limitación: era raro porque el pin no se movía, sino que tú tenías que ir moviendo la imagen. Era extraño, pero estaba bacán porque uno podía ver exactamente el lugar, aunque era fácil equivocarse porque salía como por zonas.

**Moderador:** ¿Sabes qué? A mí también me pareció que no era muy intuitivo. Yo pensé que se seleccionaba directamente el pin.

**Participante 5:** No, uno tenía que mover la imagen entera, no el pin. Moverse al lugar resultaba súper extraño.

**Moderador:** ¿Alguien más tuvo problemas con el pin? Yo la verdad nunca lo había visto.

**Participante 6**: Es que, según yo, cada pregunta debería tener instrucciones, porque al menos yo nunca había visto ese tipo de función en Kahoot.

**Moderador:** Quizás era de las últimas actualizaciones, pero me imagino que causó un poco de sorpresa. ¿Se demoraron más en esa pregunta de casualidad?

**Participante 5**: Sí, la primera vez sí; después en las otras ya no.

**Moderador:** Ah, ya. Qué bueno que se pudieron acostumbrar. ¿Qué ventajas y desventajas ven en estas tecnologías en comparación con métodos más tradicionales? Comencemos por las ventajas. ¿Qué ventajas encuentran en utilizar Kahoot u otras tecnologías frente a lo tradicional?

**Participante 1:** Yo creo que al ser más lúdico uno presta más atención y está más motivado; si fuera algo más aburrido, uno quizás no tendría tantas ganas de responder como en un Kahoot.

**Moderador:** Justo vi que el Participante 4 iba a encender el micrófono. ¿Quieres compartir algo?

**Participante 4**: Sí. Bueno, siento que ahora las tecnologías se ocupan mucho en clase, entonces tampoco es algo tan nuevo, pero la actividad en sí sirve mucho porque te obliga a poner atención a lo que estás haciendo y viendo. Eso te activa mucho más y te saca de la rutina de estar escribiendo y prestando atención pasiva, que a la larga aburre.

**Moderador:** ¿Entonces se sintieron más motivados y captó más su atención el uso de Kahoot?

**Participante 5:** Sí, además uno intentaba hacerlo lo más rápido posible para ganar. Entonces, aparte de motivarse, influye el factor competitivo, ¿no?

**Moderador**: Sí, es cierto. ¿Alguna desventaja que consideren de Kahoot?

**Participante 2**: Yo creo que una desventaja podría ser que, aunque en mi caso no sucedió, si alguien no tiene internet en ese momento o su dispositivo no funciona, queda excluido de la actividad.

**Participante 4:** Pero la universidad ofrece internet.

**Participante 2:** Sí, pero si tu dispositivo está descargado, por ejemplo, no puedes participar; a eso voy.

**Moderador:** ¿Hay alguna otra desventaja que encuentren con estas tecnologías? ¿Con Kahoot u otra plataforma de juegos digitales? ¿No? Bueno, ahora vamos a hablar un poco de la dimensión pedagógica. ¿Sintieron que su forma de aprender o de organizar el estudio cambió con estas tecnologías?

**Participante 7**: O sea, por lo menos para mí fue como una especie de diagnóstico de cómo estaba. En las preguntas que fallaba, sabía que tenía que estudiar más; entonces ahí podía ir organizando mi estudio.

**Moderador:** Y después de esto, ¿crees que Kahoot puede ser una herramienta valiosa de diagnóstico?

**Participante 7:** Sí, súper valiosa.

**Participante 6:** Perdón, al menos yo siento que esta instancia, al ser solo una, no genera tanto impacto. En cambio, si lo hiciéramos como controles de entrada o de salida de diagnóstico, sin nota, siento que sí serviría mucho. Por ejemplo, si el profesor ya lleva una hora hablando y hacemos un control de salida, podríamos integrar todas las cosas que dijo.

**Moderador:** Es cierto que, como esta intervención desgraciadamente fue de una sola vez, hubiera sido bueno evaluarla a lo largo del tiempo.

**Participante 6:** Sí, eso mismo.

**Participante 5:** Porque decir que impacte en la forma de estudio por un momento tan pequeño (de 10 minutos máximo) creo que es mucho. En cambio, si fuera algo clase a clase, creo que ahí tendría más impacto porque uno estaría más atento o motivado para que le vaya bien en el Kahoot, pondría más atención y cosas así. Pero ahora fue solo el momento, no es que cambie cómo estudio actualmente.

**Participante 3:** Perdón, como dicen, si fuera antes de la clase, con el contenido de la sesión anterior, fomentaría el estudio constante. A lo largo del semestre uno va priorizando el estudio de distintas materias dependiendo de las evaluaciones que tiene; si fuera como control de entrada o de salida, a uno lo motivaría a mantener un estudio constante de este ramo.

**Moderador:** Muchas gracias por compartir eso; qué bueno escuchar su opinión sobre la intervención. ¿En qué medida creen que estas herramientas promueven la colaboración entre compañeros en lugar del trabajo individual? ¿Sienten que el Kahoot mejora el trabajo en equipo?

**Participante 7:** Trabajo en equipo no creo que haya mucho, porque el Kahoot individual promueve la competencia entre cada uno. Creo que es algo más individual.

**Participante 3**: Sí, quizás si se hiciera en parejas o en grupos sería distinto, pero en este caso, al ser individual, se veía más la competitividad.

**Moderador:** Bueno, eso se entiende.

**Participante 5:** Sí, de todas formas, igual nos apoyábamos entre los compañeros de mesón: si alguien no respondía le soplábamos o nos preguntábamos "¿cuál respondiste tú?". Más que nada eso, pero más allá de eso, no. Todos dábamos las respuestas antes de que se acabara el tiempo para ayudar a los que no sabían.

**Participante 1:** No aporta mucho al trabajo en equipo porque, como ya dijeron, es personal; no veo cómo podría ser trabajo en equipo. Es más, de competitividad, aunque no todos lo vean así, pero claro, el que responde primero saldrá primero solo.

**Moderador**: ¿Creen que afecta de manera neutral, es decir, que no influye en la colaboración o directamente consideran que el Kahoot es perjudicial para el trabajo en equipo porque enseña a no colaborar? ¿Alguno cree que si ocuparan Kahoot más seguido se volverían más individualistas?

**Participante 5:** No creo, no creo que sea tan extremo, porque, como decía, en mi mesa había un buen ambiente. Por ejemplo, cuando alguno de nuestra mesa salía en el podio, era como "¡ah, estamos todos en el podio!". No creo que sea algo tan así, pero igual depende de los compañeros y de con quién conviva uno cerca.

**Moderador:** Siguiente pregunta: ¿participaron más, menos o igual que en las clases tradicionales? ¿Alguien consideró que participó más o se encontró más activo en esta clase? ¿Y quién participó menos? Bueno, su Kahoot fue de salida, ¿cierto? Me suena mucho que fue al final.

**Participante 5:** Fue de salida, al final de la clase.

**Moderador**: Claro, entonces es cierto que quizás no se puede evaluar la participación general en esos últimos diez minutos.

**Participante 5**: En mi caso, la clase terminó después del Kahoot, así que no afectó mucho la participación de la clase en sí.

**Moderador:** Es difícil evaluarlo entonces.

**Participante 4:** Pero, por ejemplo, podríamos verlo desde el punto de vista de que, al menos en mi sección, todos participaron en el Kahoot. No hubo nadie que se quedara fuera, así que hubo una buena participación.

**Moderador**: Claro. Por ejemplo, en las clases normales, ¿les dan casos clínicos para desarrollar o son solo clases teóricas? ¿Qué actividades suelen hacer?

**Participante 5:** En el laboratorio usamos la mesa anatómica, pasamos la materia y después reconocemos las estructuras en los fantomas.

**Moderador**: Y todos aquí, por ejemplo, ¿se dan el trabajo de buscar las estructuras y luego reconocerlas en la mesa anatómica?

**Participante 5:** Yo por lo menos sí, si no voy a estar perdida después cuando estudie; además, le saco muchas fotos a todo.

**Moderador**: ¿Ah, entonces siempre le sacan el máximo provecho al laboratorio?

**Participante 5**: Yo creo que la mayoría; por lo menos en mi sección, están todos grabando, haciendo cosas y sacando muchas fotos.

**Participante 4**: Sí, en mi sección igual.

**Moderador**: Pasemos a lo disciplinar. ¿De qué manera estas tecnologías les ayudaron a trasladar lo aprendido en la clase a situaciones de práctica clínica? Es una pregunta peculiar porque aún no tienen práctica clínica, pero ¿sienten que algo se les quedó grabado gracias a este Kahoot? ¿Nadie tuvo un sentimiento parecido?

**Participante 5**: Yo creo que sí, porque como algunas preguntas eran de reconocimiento estructural, al momento de estudiar para el control sobre eso, esa parte fue más que nada repasar: "esto es esto, esto y esto". Como fue la clase y después el Kahoot, uno aplicó de inmediato el conocimiento, lo que facilitó el estudio posterior. Por esa parte, creo que es un aporte para no tener que profundizar tanto desde cero o no estar tan perdido.

**Moderador:** Qué bueno saber que les quedó grabado algo que les servirá para el futuro. La siguiente pregunta se dirige a si las tecnologías les ayudaron a comprender mejor los contenidos de morfología.

**Participante 1:** Yo creo que sí. Por ejemplo, la mesa anatómica también es una tecnología, y si estuviéramos solo con modelos palpables, quizás no sería tan fácil distinguir todas las estructuras. En cambio, las tecnologías están súper actualizadas y uno puede ver todo detalladamente.

**Moderador:** Claro, por el alcance que tienen para practicar mejor de cara a sus evaluaciones. Centrándonos en el Kahoot, ¿hubo algo que sientan que comprendieron más después de usarlo?

**Participante 6:** O sea, no por el Kahoot en sí, pero ahí me di cuenta de las cosas que tenía que reforzar; por ese lado sí me sirvió. Respecto a lo anterior, yo nunca he aplicado el Kahoot para estudiar individualmente, pero sí he visto que en las salas de estudio a veces hay grupos grandes de personas que lo proyectan y van respondiendo Kahoots entre ellos.

**Moderador:** Qué bueno saber que han visto que puede ser una herramienta útil de estudio. Pasemos a la valoración general e impacto. Si tuvieran que aconsejar a un docente sobre en qué momento vale la pena usar estas tecnologías, ¿qué recomendaciones darían?

**Participante 3:** ¿Podrías repetir la pregunta, por favor?

**Moderador**: Sí. Si tuvieran que aconsejar a un docente sobre en qué momento vale la pena usar estas tecnologías, ¿qué recomendaciones darían?

**Participante 3**: Ahora sí. Bueno, yo creo que podría ser en un caso similar al que nos pasó, previo a nuestro primer control, ya que se asemejaba mucho a lo que era anatomía; eso nos hacía sentir un poco más seguros y nos permitía familiarizarnos con la forma de preguntar, el manejo del tiempo, etcétera. También creo que sería una buena idea hacerlo, no sé, semana por medio, ya sea de entrada o de salida, para que los estudiantes mantengan un estudio constante. Sé que en otras carreras o universidades que tienen controles de entrada y salida lo hacen principalmente con Kahoot, y eso ayuda a repasar y profundizar en la materia en general.

**Participante 5:** Yo creo que implementarlo de entrada o de salida ayuda a estudiar constantemente, porque personalmente para los controles siempre se me acumula lo visto en varias clases. Si fuera semana a semana no se acumularía tanto, sería más llevadero y generaría menos estrés a la larga.

**Moderador:** Si tuvieran que escoger que fuera de entrada o de salida, ¿cómo creen que sería mejor el Kahoot?

**Participante 3:** Yo creo que podría ser de entrada, pero enfocado en lo último que se vio, ya que daría un espacio para repasar. Si se hace al final de la clase preguntando lo mismo que se acaba de revisar, no se fomentaría el estudio fuera de la universidad, ya que lo estaríamos viendo en el momento.

**Participante 5**: Yo pensaba lo mismo: mejor de entrada, así ya estudié lo que me acaban de pasar para que me vaya mejor o para aprovechar mejor la instancia.

**Moderador:** ¿Alguien aquí no está de acuerdo con eso? ¿No? Bien. ¿Cómo se imaginan que estas tecnologías podrían evolucionar para responder mejor a las necesidades de los estudiantes de medicina en el futuro? ¿Qué esperan de Kahoot, qué le falta o cómo podría mejorar?

**Participante 3:** Como mencioné antes, creo que faltó incluir histología, porque morfología abarca tres ramas: embriología, anatomía e histología. Siento que este Kahoot estaba enfocado únicamente en anatomía. Para mejorar, podría integrar las tres ramas; en nuestro caso, todavía no entramos mucho en embriología, por eso no la nombro, pero agregar las otras áreas en Kahoot sería beneficioso.

**Moderador:** ¿Sienten que Kahoot tiene alguna limitación actualmente tal como está?

**Participante 5**: Lo mismo que decía antes: lo poco intuitivo del puntero. Además, faltaría explicar la dinámica a los que no han jugado antes, porque al inicio, si uno lee las preguntas, sabe que no van a aparecer en la pantalla del teléfono; tal vez alguien se quedó mirando solo su celular y nunca le apareció la pregunta.

**Moderador**: La siguiente pregunta dice: ¿qué aprendizajes o beneficios principales se llevaron de estas experiencias? Después del Kahoot, ¿sienten que obtuvieron algún beneficio, aprendizaje o conocimiento nuevo?

**Participante 4**: Aprender algo nuevo, no; diría más bien que sirvió para reforzar, porque al final esa era la ventaja del Kahoot: estábamos reforzando los contenidos en vez de aprenderlos desde cero.

**Moderador:** ¿Cómo describirían la motivación y el interés de los estudiantes al trabajar con estas tecnologías? En este caso, refiriéndose a ustedes mismos, ¿cómo fue su motivación e interés?

**Participante 7:** Yo diría que todos nos motivamos mucho porque fue divertido y totalmente distinto. La motivación subió y todos estaban interesados, porque fue una experiencia más agradable que una clase normal en el laboratorio.

**Participante 5**: Sí, al ser algo fuera de lo común, dejó a todos más contentos y llevó la clase a otro nivel, rompiendo la rutina de "lo mismo de siempre". Al salir, todos estábamos comentando sobre la actividad por el interés que generó.

**Moderador:** Quería tocar ese tema cuando hablábamos de la colaboración en equipo. ¿Sienten que esto mejoró su relación con sus pares? ¿Fue una experiencia en la cual compartieron con sus compañeros o se concentraron individualmente sin interactuar?

**Participante 5**: Sí, yo ya había hablado de eso; comenté que entre todos nos quedábamos hablando del tema o celebrábamos si alguno ganaba: "¡ganamos!", y cosas así.

**Moderador:** Qué bueno. ¿Y alguien no experimentó eso? ¿No? Bueno, la siguiente pregunta es: ¿qué cambiarían o mejorarían de la forma en que se aplicaron estas tecnologías? Esto ya lo hemos conversado bastante durante la reunión.

**Participante 7**: Sí, pero priorizando algo más puntual, creo que lo más importante sería la posibilidad de hacerlo más veces, porque así sería mucho más provechoso.

**Moderador:** Al final fue una experiencia gratificante que les gustaría repetir en el futuro. ¿Sienten que en esos 10 o 15 minutos que duró el Kahoot hubiera sido más valioso hacer otra cosa, o hubieran preferido una actividad diferente?

**Participante 6**: A mí me pasa que, a ese punto de la clase, ya no quiero seguir escuchando al profesor; por lo tanto, creo que es bueno que se genere esta instancia distinta para volver a captar la atención.

**Moderador**: Aprovechando ese comentario, recuerden que esto es completamente anónimo, así que no se preocupen; pueden expresarse libremente sobre el docente si lo desean.

**Participante 6:** Pero lo decía por un tema de concentración personal, no por el profesor.

**Moderador**: Se entiende, es por si alguien tenía algún pensamiento que quisiera compartir sobre la aplicación de la tecnología. ¿El docente tuvo algún problema al momento de aplicar la herramienta?

**Participante 4**: Por lo menos en mi sección todo fue fluido.

**Moderador**: Nos quedan solo unos 6 minutos. Ya no tengo más preguntas anotadas para el cierre. Obviamente, quiero agradecerles su participación; nos sirve un montón que se hayan conectado de manera puntual y que hayan respondido a las preguntas. Fueron muy participativos durante la reunión. Antes de terminar, me gustaría preguntarles si hay algo más que quisieran compartir sobre esta experiencia, ya sea positivo o negativo. ¿Nada? Bueno, por mi parte no queda más que informarles que pronto serán contactados si necesitamos alguna validación de los resultados. Nos mantenemos en contacto; de nuevo, muchas gracias por conectarse, en especial a esta hora. Pueden desconectarse cuando gusten.

**Participantes**: Muchas gracias.

## TRANSCRIPCIÓN FOCUS GROUP 2

Versión Revisada Fecha: Viernes 3 de octubre de 2025 (19:00–20:00) Moderadora: Valentina Riquelme Vilches

Proyecto: Raíces 2 – Uso de Kahoot en clases de Morfología

Introducción

**Moderadora**: La idea es que esta sea una instancia para dialogar y conversar. Yo estaré como moderadora, pero ustedes son los protagonistas.

Mi nombre es Valentina Riquelme, formo parte del grupo de investigación “Raíces 2”, junto a Sofía y Eduardo, a quienes probablemente ya conocen por el consentimiento informado.

Sofía es nuestra líder y yo pertenezco a la sede de Santiago.

Esta actividad busca generar un espacio de diálogo dividido en distintas etapas: preguntas iniciales, dimensiones tecnológicas, pedagógicas y disciplinares, además de una valoración general sobre el impacto de la tecnología aplicada —en este caso, el uso de Kahoot!.

Les agradezco mucho por participar, especialmente siendo viernes y después de ayudantía. Sabemos que no es fácil.

Recuerden que aquí no hay respuestas buenas ni malas; la idea es compartir libremente las experiencias y opiniones.

Preguntas de inicio Moderadora:

Para comenzar: al pensar en la clase donde se utilizó Kahoot, ¿qué es lo primero que recuerdan de esa experiencia?

**Participante** 2:

Recuerdo que fue cerca del primer control. Sentí algo de nerviosismo porque se parecía mucho a lo que sería ese control.

**Moderadora**:

Claro, tiene sentido. ¿Alguien más comparte esa impresión? Participante 4:

Sí, opino igual. Fue una buena forma de evaluarnos de manera distinta, más didáctica, para ver si estábamos preparados o si nuestro método de estudio era el correcto.

**Participante** 7:

Para mí fue una experiencia simpática. Me recordó al colegio y me pareció positiva, sobre todo porque ayudó a bajar los nervios antes de la primera evaluación.

**Participante** 5:

Yo la encontré una actividad muy didáctica. Permitía medir nuestro nivel y practicar con tiempo cronometrado, lo que fue excelente antes del primer control.

**Moderadora**:

Perfecto. Pasemos a la siguiente pregunta: ¿cómo describirían su primera impresión al usar tecnologías como Kahoot en asignaturas clínicas, como Morfología?

**Participante** 1:

No era la primera vez que usábamos Kahoot, pero me pareció útil en Morfología. Normalmente trabajamos con la mesa anatómica o con imágenes similares a las de los controles. Kahoot permite medir no solo el conocimiento, sino también el manejo del tiempo. A veces lo más difícil no es reconocer la estructura, sino hacerlo rápido, y Kahoot te cronometraba, lo que lo hace muy relevante.

**Participante** 5:

Comparto totalmente la opinión de la participante 1. Moderadora:

Y con respecto a sus expectativas: ¿qué pensaban antes de usar Kahoot y cómo cambiaron después de la experiencia?

**Participante** 6:

Al principio pensé que Kahoot era algo más infantil, como un juego fácil. Pero me di cuenta de que depende mucho de cómo se utilice. En Morfología, y en general en cualquier materia, puede hacerlo todo más didáctico y entretenido si se usa correctamente. Cumple perfectamente con esas funciones.

**Participante** 7:

Yo tenía dudas sobre cómo se aplicarían las preguntas en Morfología, pero al usar Kahoot vi que eran muy parecidas a las de los controles. Sirvió mucho para relajarme.

Dimensión tecnológica Moderadora:

Pasemos ahora a la parte tecnológica. ¿Qué obstáculos técnicos enfrentaron durante la actividad y cómo los resolvieron?

**Participante** 6:

En realidad, ninguno. Es muy sencillo: ingresas el código o escaneas el QR. Si alguien no tiene internet, puede usar el wifi de la universidad. En mi grupo nadie tuvo problemas.

**Participante** 1:

Por mi parte tampoco. Si se refiere a conexión o manejo del sistema, no hubo inconvenientes. Solo recuerdo una pregunta con una palabra distinta a la que usamos comúnmente para una estructura anatómica, pero eso fue más un tema de aprendizaje que técnico.

**Participante** 2:

Igual que mis compañeros, no tuvimos problemas con la conexión ni con la aplicación. Moderadora:

¿Y qué funciones les resultaron más útiles o novedosas? ¿Cuáles creen que tuvieron un impacto más limitado?

**Participante** 2:

Lo que más me llamó la atención fue tener que colocar un PIN en la estructura que se indicaba. Nunca se me habría ocurrido, y lo encontré muy útil.

**Participante** 4:

Sí, el PIN también me llamó la atención. Participante 1:

Yo no sabía que en Kahoot se podía usar un PIN. Pensaba que solo era posible en otras plataformas, como Mentimeter. Me pareció interesante.

**Moderadora**:

¿Y hubo algo que les resultara más confuso o menos útil? Participante 1:

En mi caso, una pregunta sobre la uretra tenía un término distinto al que solemos usar, y eso me confundió un poco.

**Moderadora**:

Ahora, ¿qué ventajas y desventajas ven en el uso de Kahoot en comparación con métodos más tradicionales?

**Participante** 6:

Más que ventajas o desventajas, creo que Kahoot complementa muy bien las clases tradicionales. Tener una clase y luego aplicar una actividad interactiva ayuda a reforzar lo aprendido de forma atractiva y entretenida.

**Participante** 1:

Una posible desventaja es que puede generar cierto desorden al inicio, cuando todos se conectan o compiten. Además, al tener un componente competitivo, algunos pueden desmotivarse si no quedan entre los primeros lugares.

**Participante** 7:

A mí me pareció muy didáctico, sobre todo porque ayuda a integrar el contenido de forma más sencilla. Personalmente, me sirvió mucho como método de estudio.

**Moderadora**:

Perfecto. Vamos a continuar con la siguiente dimensión. Dimensión pedagógica

**Moderadora**:

¿Sintieron que su forma de aprender o de organizar el estudio cambió con estas tecnologías?

**Participante** 1:

En mi caso, no. Kahoot fue más bien un complemento, pero no cambió mi método de estudio. Me sirvió para poner a prueba lo que ya había aprendido.

**Participante** 2:

Tampoco cambió mucho mi manera de estudiar, pero me ayudó a responder más rápido en evaluaciones como la gymkana. No se me habría ocurrido practicar de esa forma sin Kahoot.

**Participante** 6:

Creo que aún no está completamente incorporado en nuestra rutina. Si después de cada clase se aplicara un Kahoot, entonces sí podría decir que influye en la forma de estudio.

**Participante** 4:

Coincido con mis compañeros. No cambió mi método, pero sí me sirvió como una forma de evaluación previa, para comprobar si realmente había aprendido.

Colaboración y trabajo en grupo Moderadora:

¿Creen que estas herramientas promueven más la colaboración entre compañeros o el trabajo individual?

**Participante** 1:

Podría ser útil si se aplicara por grupos o mesones, así se pondrían a prueba los conocimientos de manera colectiva.

**Participante** 6:

En mi opinión, no promueve tanto el trabajo en grupo. Es más una competencia individual, aunque sea divertida. Compites con tus amigos, pero no trabajas con ellos.

Participación

**Moderadora**:

¿Sintieron que participaron más, menos o igual que en una clase tradicional? Participante 4:

Yo participé más, porque me cuesta mantener la atención durante las clases, y con Kahoot logré enfocarme mejor.

**Participante** 5:

Participé más también. El anonimato ayuda a perder el miedo a equivocarse. Participante 2:

Sí, eso mismo. Al ser anónimo, uno se atreve más a responder sin miedo. Participante 1:

Yo participo bastante en clases, pero con Kahoot a veces me frustro cuando me equivoco por apuro o por confusión con los colores de las respuestas. En esos casos, dejo pasar una pregunta y luego sigo.

Dimensión disciplinar

**Moderadora**:

¿De qué manera estas tecnologías les ayudaron a trasladar lo aprendido en la clase a situaciones prácticas o clínicas?

**Participante** 2:

Creo que ayudan a reconocer mejor las estructuras, incluso sin tener la mesa anatómica, aplicando el conocimiento en distintos contextos.

**Participante** 6:

No entendía bien lo de “práctica clínica”, pero si se refiere al laboratorio, sí. Kahoot ayuda a consolidar lo aprendido en clase mediante las preguntas.

**Participante** 7:

Estoy de acuerdo. Sirve para reforzar conocimientos y aplicarlos mejor en otras instancias, como las prácticas.

**Moderadora**:

¿Estas tecnologías les ayudaron a comprender mejor los contenidos de Morfología?

**Participante** 1:

Sí. En Kahoot uno ve las estructuras desde distintas perspectivas: en modelos físicos, en la mesa anatómica y en fotos. Eso obliga a desarrollar una mirada más completa y adaptable.

**Participante** 5:

De todas maneras, al responder preguntas con tiempo limitado, uno se prepara mejor.

**Participante** 6:

No me ayudó a comprender nuevos contenidos, pero sí a consolidar los conocimientos previos.

**Participante** 4:

Comparto esa opinión. Kahoot no enseña cosas nuevas, pero permite confirmar que realmente aprendimos lo visto en clase.

**Participante** 2:

Exacto. Más que aprender, sirve para reforzar lo estudiado. Valoración general e impacto

**Moderadora**:

Si tuvieran que aconsejar a un docente sobre cuándo usar Kahoot, ¿qué recomendarían?

**Participante** 6:

Lo recomendaría al 100%. Idealmente al final de cada clase, con 10 o 15 minutos para hacerlo. También sería útil revisar las respuestas y explicar por qué una opción es correcta.

**Participante** 1:

Creo que es mejor usarlo después de haber visto toda la materia y hecho un repaso general. Si se aplica justo al final de una clase muy cargada, puede generar más competencia que aprendizaje.

**Participante** 5:

De acuerdo, debería usarse antes de un control, como una forma de autoevaluación.

**Participante** 3:

Sería bueno aplicarlo más seguido, porque es una actividad dinámica.

**Participante** 4:

Lo ideal sería tener un Kahoot al final de cada clase, aunque sé que eso requiere tiempo para prepararlo. Ya aplicarlo antes de los controles sería una gran ayuda.

**Participante** 1:

También podría organizarse por sistemas o temas específicos, para no mezclar todo. Así se aprovecharía mejor.

**Moderadora**:

¿Cómo se imaginan que estas tecnologías podrían evolucionar para responder mejor a las necesidades de los estudiantes de Medicina en el futuro?

**Participante** 6:

Sería genial que Kahoot fuera más fácil de crear. Tal vez que la universidad tuviera un banco de Kahoots por curso, con preguntas estandarizadas que los estudiantes pudieran practicar libremente.

**Participante** 2:

Sí, lo que dijo el participante 6 sería muy útil. Facilitaría mucho la práctica y reduciría la carga de los profesores.

**Moderadora**:

¿Qué aprendizajes o beneficios principales rescatan de esta experiencia?

**Participante** 1:

Kahoot permite ponernos a prueba y medir cuánto aprendimos, además de manejar el factor emocional del tiempo y la presión, similar a un control.

**Participante** 7:

A mí me ayudó a saber si estaba estudiando bien. Fue una forma de autoevaluación y de simular la presión de una evaluación real.

**Participante** 6:

Lo veo como una progresión útil: primero Kahoot, luego un control preparativo y después la evaluación real. Ayuda a reducir el estrés.

**Participante** 3:

Sirve para medir conocimientos y acostumbrarse al tiempo, como si fuera un control real. Motivación e interés

**Moderadora**:

¿Cómo describirían la motivación y el interés de los estudiantes al trabajar con Kahoot?

**Participante** 4:

La principal motivación es la competencia sana con los compañeros. Da ganas de participar y de superarse.

**Participante** 6:

Sí, concuerdo. Competir con los amigos hace la clase más entretenida y fomenta la interacción.

**Participante** 2:

Además, hace que la clase sea más dinámica. No es solo escuchar al profesor, sino participar activamente.

Mejoras posibles

**Moderadora**:

Si pudieran mejorar algo en la aplicación del Kahoot, ¿qué cambiarían?

**Participante** 2:

Nada, me pareció perfecto hacerlo al final de la clase. Fue relajado y bien organizado.

**Participante** 1:

También pienso que estuvo bien aplicado y abarcó los temas relevantes para el control.

**Participante** 6:

De acuerdo. Dependiendo de la materia, podrían variar el número de preguntas o el tiempo, pero en general fue adecuado.

**Participante** 1:

Solo sugeriría incluir más imágenes de la mesa anatómica, porque a veces la vista o el ángulo hacen que sea más difícil identificar las estructuras.

**Participante** 5:

Yo lo dejaría igual.

**Participante** 3:

Podría ser un poco más largo y con preguntas más complejas, pero estuvo bien para ser el primero.

**Participante** 1:

También se podrían combinar modelos y fotos de la mesa en un mismo Kahoot, especialmente antes de las gymkanas, donde se usan ambos.

**Participante** 7:

Sí, depende de la instancia. En los controles se usa más la mesa, pero en las gymkanas ambos tipos de material.

Cierre del focus group

**Moderadora**:

Antes de terminar, ¿qué les pareció esta instancia de conversación?

**Participante** 1:

¿Esto tiene un fin investigativo?

**Moderadora**:

Sí, pertenece al grupo de investigación Raíces 2 de la universidad. Estamos analizando el uso de tecnologías educativas en distintas asignaturas, como Morfología, Semiología y Cirugía.

**Participante** 1:

Ah, perfecto. Gracias por aclararlo.

**Moderadora**:

¿Alguno tiene comentarios sobre la experiencia o sugerencias adicionales?

**Participante** 1:

Sí, como sugerencia más tradicional, quizá podrían entregar un material de apoyo para los laboratorios, como un PPT o una hoja resumen con los términos más importantes. A veces uno tiene que revisar la grabación para repasar, y sería útil tener una guía escrita.

**Moderadora**:

Buena idea, muchas gracias.

**Participante** 2:

Yo creo que la instancia fue muy buena. Ojalá se repita más seguido.

**Moderadora**:

Gracias a todos por participar, especialmente un viernes. Aprecio mucho su disposición y opiniones, que son fundamentales para mejorar el aprendizaje y la enseñanza. ¡Les deseo mucho éxito en Morfología y en lo que viene!

(Todos se despiden)

## TRANSCRIPCIÓN ENTREVISTA DOCENTE: viernes 10 de octubre del 2025.

**Moderadora**: Ya, creo que ahí estamos grabando. Bueno, primero agradecerle por su participación. Ya con esto cerraríamos su intervención, así que…

**Profesor**: De nada, se agradece que me hayan considerado.

**Moderadora**: Vamos a comenzar. Primero, en la dimensión general: ¿Cómo describiría usted su experiencia al aplicar Kahoot! en la clase de morfología?

**Profesor**: Fácil y dinámica. Fácil en el sentido de que, si bien no estaba habituado a utilizarlo como herramienta, es bastante intuitivo, cómodo y familiar. No fue nada complicado; sí, obviamente, tuve que habituarme un poco, pero la forma de aplicarlo y llevarlo a cabo fue muy sencilla. Y dinámica porque los chicos inmediatamente se mostraron interesados. Se transformó en algo muy natural para ellos, sintieron el deseo de hacerlo sin necesidad de convencerlos o fomentar que participaran. Lo tenían muy internalizado.

**Moderadora**: La segunda pregunta es: ¿Qué expectativas tenía respecto a la reacción de los estudiantes y cómo se compararon con lo que observó?

**Profesor**: Yo esperaba que iban a ser así de entusiastas, así que en ese sentido estuvo más que cumplido. Lo que más me sorprendió fue que la reacción fue inmediata: casi no hubo que explicar, solo se sorprendieron de que había un Kahoot!, y de inmediato lo empezaron a ocupar.

**Moderadora**: ¿Le resultó sencillo o complejo aplicar la plataforma en la práctica?

**Profesor**: Sencillo. Es bien amena la interfaz que tiene la aplicación. No sé si podría decir lo mismo si se proyectara desde otro dispositivo, pero al menos desde el computador al monitor fue muy fácil.

**Moderadora**: ¿Detectó alguna dificultad técnica en el momento de la clase?

**Profesor**: No. A pesar de esta alternativa nueva de ubicar el PIN, no hubo ningún problema técnico. No cabría decir que hubo algo así.

**Moderadora**: ¿Considera que Kahoot! facilitó o complicó la dinámica de cierre?

**Profesor**: No la complicó. Como elemento final de medición o profundización, o para ver la interiorización de los contenidos, sí fue una herramienta adecuada. Facilitó incorporar los conocimientos.

**Moderadora**: ¿Qué tan intuitiva fue la plataforma para usted en comparación con otras herramientas digitales que ha visto en docencia?

**Profesor**:No tengo mucho manejo con herramientas de ese tipo, pero eso mismo me sorprendió: a pesar de no ser muy habituado, fue bastante simple seguirla. Ya fuera desde un aparato móvil o desde el computador, era fácil guiarse a través de la cuenta y la biblioteca, con un preset ya listo. Eso ayuda mucho si ya hay un pool de dinámicas establecido. El diseñar un Kahoot! puede ser otro tema, pero aplicarlo es sencillo.

**Moderadora**: ¿Cómo percibió la participación y motivación del estudiantado durante la actividad?

**Profesor**: Muy	abierta. Se entiende por qué se ocupa tanto: da una dinámica de juego y el sentido de competición fomenta que sea algo más “jueguil”. Eso hizo que todos estuvieran dispuestos	y	con	ganas. Termina siendo una forma de aprender de manera más lúdica, casi “soslayada”, pero efectiva.

**Moderadora**: ¿Observó diferencias en la interacción de los estudiantes en comparación con una clase sin esta herramienta?

**Profesor**: En mi caso estoy un poco limitado porque no suelo ocupar herramientas al final de la clase, pero sí, hubo diferencias claras. La actividad fue mucho más lúdica, más activa y conectada que, por ejemplo, pasar solo una guía con una imagen. Hacer lo mismo mediante una guía en papel, además de ser menos sustentable, genera menos interés. En cambio, Kahoot! facilitó mucho más la motivación del grupo. Además, el hecho de que todos participaran al mismo tiempo y al mismo ritmo hizo que la actividad fuera más rápida y diligente que trabajar únicamente con guías.

**Moderadora**: ¿Cree que la herramienta aportó a reforzar los aprendizajes esperados?

**Profesor**: Sí, creo que sirve como herramienta para medir qué tan logrados están los aprendizajes esperados. Respecto a reforzarlos, depende mucho del tipo de imágenes utilizadas, pero en general contribuye a verificar si los contenidos están entendidos, si falta algo o si es necesario reforzarlo de otra forma. En términos generales, sí aporta.

**Moderadora**: ¿Consideró que Kahoot! permitió dar retroalimentación más inmediata a los estudiantes en comparación con otros métodos?

**Profesor**: Sí. La plataforma permite entregar retroalimentación inmediata gracias al tiempo entre preguntas y al sistema de puntaje y premiación. Habría que buscar la forma de que el refuerzo entre cada pregunta sea más natural, pero aun así facilita la retroalimentación inmediata. Los estudiantes reconocen sus aciertos, y el tiempo entre preguntas permite entregar una respuesta o guía más específica y concisa respecto a lo que se esperaba lograr.

**Moderadora**: Ok. ¿En qué medida Kahoot! ayudó a los estudiantes a comprender mejor los contenidos de morfología?

**Profesor**: Primero, ayuda a identificar de inmediato si reconocen las imágenes o los contenidos. Eso permite ver si aprendieron lo necesario y, a partir de ahí, aclarar dudas. Además, al generar interés, también genera ganas de estudiar. En una asignatura tan visual como morfología, el estímulo es constante: querer ver, esperar la imagen siguiente y darse cuenta si necesitan reforzar algo o si ya entendieron lo que se pretendía.

**Moderadora**: ¿Hubo aspectos del contenido que, según su percepción, no lograron transmitirse bien mediante esta dinámica?

**Profesor**: Diría que sí, pero en un ámbito extraoficial. La herramienta depende de una buena proyección y de imágenes adecuadas para lograr las temáticas. Eso es algo a mejorar, pero es circunstancial. No es un limitante; al contrario, permitió que los estudiantes se familiarizaran con los contenidos en vez de percibirlos como algo distante o enajenado, como podría ocurrir en una clase tradicional. Solo hay que cuidar el diseño del material, pero eso es común a todas las herramientas educativas.

**Moderadora**: Muy bien. ¿Cómo valoraría la integración de la herramienta con la clase en términos pedagógicos y disciplinares?

**Profesor**: Si se pudiese usar, sería un plus para la clase. No veo razón para que no funcionara. Requiere cierto tiempo de organización, diseño e implementación, pero puede integrarse sin problemas. Quizás no en todas las clases, para no perder la dinámica, pero sí de forma más rutinaria.

**Moderadora**: ¿Considera que Kahoot! generó un aporte real al aprendizaje en esta asignatura?

**Profesor**: Sí. Si los estudiantes están motivados, y la herramienta es lúdica pero con contenido académico, eso siempre ayuda al aprendizaje.

**Moderadora**: ¿Le gustaría repetir o adaptar esta experiencia en el futuro?

**Profesor**: Sí, me gustaría. Intentaría hacerlo con un diseño más profesionalizado en cuanto a las imágenes o con algo más conciso. Si el contenido es muy extenso o disperso, cuesta focalizar. Idealmente sería algo temático y puntual para pulir mejor los contenidos. Así serviría no solo como repaso o medición, sino también como una herramienta para presentar conocimientos más acotados y específicos.

**Moderadora**: Ok, y para cerrar. Luego de esta experiencia, ¿le interesaría seguir incorporando distintas herramientas o tecnologías educativas en sus clases de morfología? Y, de ser así, ¿por qué?

**Profesor**: Bueno, ya tenemos un elemento tecnológico dentro de todo. Pero claro, siempre que sea en pro de la asignatura y ayude a representar o dar a conocer de mejor manera lo que se quiere enseñar, obviamente están las ganas de utilizar nuevas herramientas. En este caso, conocí Kahoot! desde el perfil de una herramienta para medir la integración de contenido al final de la clase. Y al analizarlo, veo que también podría usarse para fomentar el estudio, para profundizar o para plantear nuevas incógnitas más allá de lo visto. Esas posibilidades son más que bienvenidas. Creo que sí tiene las capacidades para ello. Solo hay que aprender a utilizarlo y “domesticarlo” un poco más.

**Moderadora**: Bueno, esas eran todas mis preguntas. Damos por finalizada su entrevista docente y le agradecemos nuevamente por su participación. Puede desconectarse. Nos vemos.

**Profesor**: Gracias.
